# Preventing Knowledge Hiding Behaviors Through Workplace Friendship and Altruistic Leadership, and Mediating Role of Positive Emotions

**DOI:** 10.3389/fpsyg.2022.905890

**Published:** 2022-06-23

**Authors:** Ying He, Xiaoying Wei

**Affiliations:** School of Business, Nanjing University, Nanjing, China

**Keywords:** knowledge hiding, workplace friendship, leadership development, positive emotions, performance management

## Abstract

Studies related to knowledge hiding prevention are limited and need attention. Hence, the present study attempts to measure the direct impact of workplace friendship and altruistic leadership on preventing the knowledge hiding behavior; and also, in the presence of positive emotions. The study has also checked the mediating role of positive emotions in these relationships. The target population of the study is the employees working in the government sector (sample size of 496). The present study has employed quantitative research techniques for testing the hypotheses. Smart-PLS 3 software has been employed to run the partial least square structural equation modeling. Findings of the study have given major indications about the positive role of workplace friendship and altruistic leadership in preventing the hiding of knowledge among employees. It has also been revealed that positive emotions play a significant role in augmenting the relationship of workplace friendship and altruistic leadership with knowledge hiding behavior. This study adds a significant contribution to the body of knowledge by measuring the mediating role of positive emotions in decreasing the knowledge hiding behavior in the presence of workplace friendship and altruistic leadership.

## Introduction

Knowledge is a key instrument for enhancing business performance, as today's businesses recognize it. In this regard, researchers try to identify the most effective strategies to properly manage knowledge (Gagné et al., 2019). There are certain factors (e.g., overall organizational environments, governance, socializing culture, and disagreement), intrinsic motivation (e.g., approach toward KS and belief), and personal attributes (e.g., psychological ownership and self-efficacy beliefs) which affect knowledge sharing (KS). Having a comprehensive awareness about improving KS, professionals continue to engage in knowledge management activities. This is to reduce the impact of the negative behavior of employees who try to hide knowledge from their fellows in organizations (Peng, 2013; Kim et al., 2017; Bavik et al., 2018).

Knowledge hiding (KH) is incomparable to the growing interest in KS research. Establishing measures to discourage organizational members from engaging in KH conduct and back them up to share the knowledge with their colleagues is one of the most difficult tasks in knowledge management (Peng, 2013). The failure to pay heed to KH is due to the erroneous notion that KS and KH are on different sides of the same spectrum (Khalid et al., 2018). According to newer research, KS and KH are distinct approaches that do not exist on opposite sides of the same spectrum (Connelly et al., 2019). The reasons for the absence of KH and KS are remarkably different. People who don't even share their knowledge are motivated by a desire to purposely withhold their knowledge whenever asked by their coworkers (Connelly et al., 2019).

Circumstances that encourage KS may not apply to KH behavior (Serenko and Bontis, 2016). Organizational people who exhibit KH behavior also demonstrate an attempt to control and manage their knowledge supposedly due to selfishness against their counterparts and the enterprise (Rhee and Choi, 2017). It is a need for time to explore ways that help in preventing such behaviors at an organizational level. Therefore, researchers have to find the drivers of KH which inhibit the employees from sharing knowledge among colleagues (Abdillah et al., 2020). Many researchers have taken KH as an effort for concealing and holding knowledge deliberately when asked by others (Bari et al., 2019). To operationalize the current research, the authors take KH as a behavior that is prevalent in employees who deliberately hide knowledge from each other. Since KH is a sort of behavior of the employees, it was proposed in research that behaviors are molded through leadership in an organization (Yukl et al., 2019).

In some other research, it was assessed that within an organization, leadership style is crucial in defining subordinates' work behavior (Inceoglu et al., 2018). During the last few years, most leadership studies have focused on identifying features of behavior that explain how a leader may improve the working behaviors of the employees in a team at work. A great number of behavioral research has been undertaken, however, the quantity and types of behaviors evaluated vary significantly. Most leaders directed behavior research only looked at a few behaviors from an accepted theory like transformational leadership or a Leader Behavior Description questionnaire. The considerably smaller amount of research looked at a few particular types of leadership, but they seldom encompassed the whole spectrum of important activities associated with the behaviors of employees (Yukl et al., 2019).

Most of the research has been constrained by its dependence on broad definitions of leadership, which has hampered progress in enhancing our knowledge of successful leadership and strategies to improve. The studies on widely defined behaviors indicated that results were directed toward research on components of broad behaviors (Yukl et al., 2019). Therefore, leadership has been found connected with the behaviors of employees which is a psychological approach to improving the efficiency of the employees. Knowledge hiding is a behavioral approach of the employees in an organization regarding their work but very scarce literature is available on the behavioral changes in employees through leadership. Earlier studies have used oppressive support and coaching to understand why employees conceal their knowledge, yet particular styles of leadership which may prevent KH behavior in followers are still to be investigated (Feng and Wang, 2019).

Therefore, the current study looks into a form of constructive leadership known as altruistic leadership. Such leadership aims to prevent KH behavior among employees. Altruistic leadership, also known as human-centered leadership, pertains to a leader's activity that demonstrates the unselfish compassion of employees by prioritizing the employees' interests over his own (Salas-Vallina et al., 2018). Altruistic leadership, in contrast to other human-centered leadership styles (such as inspirational, genuine, moral, and servant leadership), emphasizes great attention to employees' needs and sacrifices self-interest to benefit employees. By drawing on their subordinates' ideals and emotions, altruistic leaders seek to motivate their subordinates to develop their dedication and passion. Subordinates will see such behavior as “putting aside” their interests to work successfully for the larger good of the organization (Salas-Vallina and Alegre, 2018). Altruistic leadership is critical in generating positive behaviors among employees, including job happiness, interpersonal humor, and inventiveness. Furthermore, it is unclear how this sort of leadership eliminates detrimental tendencies like KH (Salas-Vallina et al., 2018).

Altruistic leaders treat their employees with modesty, compassion, empathy, respect, and love. Leaders pay attention to their subordinates' thoughts and needs, express sympathy, offer coaching, training, and support when needed, and maintain a friendly demeanor (Anderson and Sun, 2017). This research makes a substantial contribution to the concepts of leadership and KM by investigating and proposing a theoretical basis that ties altruistic leadership to the behavior of KH. In contrast to a previous study that investigated how abusive supervision can promote KH behavior (Khalid et al., 2018), the present study analyses how altruistic leadership can operate as a preemptive facilitator in preventing KH behavior among organizational members. The current research is the first to look at altruistic leadership as a strategy for preventing KH behavior.

The authors take altruistic leadership as a useful and effective experience for employees in which improvement and well being of them are considered a goal. According to Dasborough (2006), employees' emotive experiences in the workplace are triggered by leadership style. As a result, leaders' selfless behavior may elicit positive emotions among employees. Positive emotions are previously defined as situational responses which are desirable and pleasant and range from contentment to joy (Lundqvist and Kenttä, 2010). Based on this elaboration, the authors take positive emotions as the pleasant response of employees when given a friendly atmosphere and leadership. Earlier research has discovered that positive emotions induced by a leader's behavior affect certain social behaviors including organizational citizenship (Goswami et al., 2016). Moreover, it's uncertain if leader-evoked positive emotion indirectly influences the interaction between altruistic leadership and bad behavior like KH. Therefore, to fill this gap, the current study utilized positive emotion as a mediator between altruistic leadership and KH behaviors.

Another aspect of the current study was the role of workplace friendships which may prevent KH behaviors among workers. It is defined as a friendship that helps in improving commitment, common interests, shared values, and trust among employees in the workplace (Hsiao-Yen et al., 2009). In this paper, the authors consider workplace friendship as a tool for developing strong ties among employees to prevent negative behaviors which are detrimental to organizations. Cerne et al. (2014) argued that a lack of trust and connectedness amongst employees leads to knowledge hiding, which harms work engagement. The conclusions are backed up by some other research published by Butt (2019); Butt and Ahmad (2019), which claims that hiding knowledge pushes knowledge searchers to leave. The causes and repercussions of KH have been well-studied in academia, but it is uncertain how businesses may reduce knowledge hiding. According to some recent studies, the existence of a workplace friendship between managers can significantly reduce KH behavior (Butt, 2018, 2019; Butt and Ahmad, 2019; Hernaus et al., 2019).

Employees who rely on workplace friendships to advance the firm's interests, according to Butt and Ahmad (2019), are usually more honest with each other. This approach helps in inhibiting harmful work attitudes like KH and knowledge holding. Similarly, Arain et al. (2020) claimed that businesses might promote the employees to establish workplace friendships because such ties typically result in significant devotion, and that can be a useful deterrent to KH behavior. According to a new study, while KH is a serious problem in many businesses, it is feasible to mitigate its harmful impacts if managers cultivate personal relationships (Noor et al., 2020). The authors further contend that employees who work together and know each other personally are more willing to assist one another in difficult situations.

Consequently, individuals may be hesitant to participate in KH behavior. Unfortunately, all of this is based on hearsay, and there is no experimental evidence to back up these statements. Companies and organizations have to bear huge losses due to this KH behavior. Moreover, no attempt has been made to reveal what businesses may do to reduce knowledge hiding (Butt et al., 2021a). To fill this gap, current research has focused on the roles of altruistic leadership and workplace friendships in preventing or minimizing the negative effects of KH behaviors in organizations. This research tried to answer a few questions given below.

- RQ1. What is the possible role of altruistic leadership in preventing KH behavior?
- RQ2. How can workplace friendships minimize the detrimental role of KH behavior?
- RQ3. How positive emotions of employees can facilitate the prevention of KH behaviors in an organization?

These questions are addressed by assessing the relationships between altruistic leadership, workplace behaviors, positive emotions, and KH behaviors.

## Theoretical and Hypothesis Support

This research is backed by some theories which could provide the basis for trickling down the effects of leadership on preventing KH in the employees. For this purpose, research gets support from social learning theory Bandura (1977) along with social exchange theory (Blau, 1964). The leaders are considered role models for the workers, and they try to learn from them. These leaders provide their stature as models. It implies that workers should learn acceptable and desirable behaviors in their workplaces primarily through modeling. Such active learning entails leaders, especially those in power positions, such as team leaders, first witnessing and then copying their behaviors (Liden et al., 2013). Prior research has shown that workers view their leaders as positive examples, trainers, and information sources from whom employees learn and copy their leaders' behavior, which supports these ideas (Ling et al., 2016).

Because previous research has shown that employees tend to emulate their leaders' favorable and unfavorable actions through social learning processes, this study focused on how to minimize knowledge hiding through trickling down the leadership effects. Moreover, this process also involves a social exchange between leaders and employees. When leaders are truly dedicated to their organizations, they tend to motivate their employees who in turn would improve their performance and avoid negative behaviors which could deteriorate their performance. Leadership in this way tries to improve the performance of employees and more specifically their performance improves if they avoid knowledge hiding among themselves. Modeling as a crucial source through which workers learn acceptable behavior by witnessing and replicating their role model's acts, particularly those in leadership positions, such as managers and supervisors, distinguishes social learning theory from other learning reward theories (Arain et al., 2022).

A rising number of leadership studies have employed social learning models to determine the transmission of leadership principles, beliefs, and behaviors between organizational members, based on the dynamics of social learning theory (Ling et al., 2016; Arain et al., 2022). This study focused on a specific type of leadership, which is altruistic leadership. This kind of leadership gets support from some theories based on other kinds of leadership. Previous studies on effective leadership did not pay much attention to leader values and integrity, but interest in them has grown in recent years. In theories of spiritual leadership and servant leadership theory, values such as integrity, altruism, empathy, justice, fortitude, and humility are emphasized (Greenleaf, 2002; Avolio and Gardner, 2005). According to proponents of these theories, leaders whose actions represent these principles will be more efficient. Drawn on these theories, altruistic leadership could provide empathy to their employees which would ultimately prevent them from KH.

### The Negative Association Between Workplace Friendship and Knowledge Hiding

Workplace friendships are distinct from social connections that exist on a personal level but are restricted to corporation knowledge. In addition, previous research reveals that effective professional friendships are marked by dedication and exchange, as well as the sharing of important company knowledge and a stronger level of trust and dedication from partners (Butt, 2019). Friendships in the workplace are crucial to a company's success. Leaders with personal relationships within inter-firm collaborations, for instance, are more efficient, motivated, and creative, leading to more turnover for their organizational units (Fehlings, 2020). Furthermore, there is substantial evidence that friends make better business partners because they are more trustworthy, loyal, and dedicated, which can have a positive impact on their commercial relationships (Butt, 2019).

The critical function of information exchange in an organization has recently grown even more critical, gaining increased attention from academics (Bouncken and Kraus, 2021). In contrast, while many firms want their employees to be involved in sharing their expertise with colleagues, many decide to keep their information hidden when requested for help (Rao et al., 2021). The goal of this study is to see if workplace friendship harms employee knowledge hiding (KH) (Rhee and Choi, 2017). Today's organizations understand that knowledge is a critical tool for improving company success (Commodari and La Rosa, 2020). As a result, academics are attempting to determine the most effective techniques for effectively managing information.

One of the most challenging contests in knowledge management is establishing mechanisms to dissuade organizational members from engaging in KH behavior while also encouraging them to share information with other individuals (Chen et al., 2022). When colleagues seek assistance, employees may choose to hide or keep information for a specific reason, which is referred to as knowledge hiding (Serenko and Bontis, 2016). Such behavior has a detrimental influence on an organization's employees' innovation and organizational work performance and even the performance of new product project team members. It can be safely claimed that workplace friendships could lead to better performance of an organization. Strong friendships are the major obstacle in KH. Therefore, based on this literature, authors assumed that such friendships could help in preventing knowledge hiding.

**H_1_:** *Workplace friendship negatively impacts KH*

### Altruistic Leadership Has a Negative Association With Knowledge Hiding

Knowledge hiding is not a comprehensive collection of undesirable behaviors. Knowledge hiding, unlike unproductive workplace behaviors, is not always designed to harm an organization to achieve its objectives. Instead, it is a typical reaction to a certain scenario. In contradiction to other human-centered leadership styles (such as inspiring, real, moral, as well as servant leadership), the altruistic leadership style is centered tremendous attention on workers' needs and self-interest sacrificed for the benefit of employees (Mostafa and Bottomley, 2020). Altruistic leaders try to urge their employees to grow their devotion and passion by relying on their principles and emotions (Belwalkar et al., 2018). Altruistic leadership is essential for instilling beneficial behaviors in employees, such as job satisfaction, interpersonal humor, and innovation (Men, 2021). Additionally, it is questionable how this type of leadership avoids negative tendencies like knowledge hiding (Noor et al., 2020).

Altruistic leaders show sincerity, compassion, understanding, respect, as well as affection for their staff. According to new research, altruistic leadership is crucial in boosting employees' optimistic perceptions, such as job contentment (Salas-Vallina and Alegre, 2018). Furthermore, an altruistic leader's likable demeanor can create a comfortable and cheerful environment, which affects subordinates' positive attitudes. Salas-Vallina and Alegre (2018) discovered that altruistic leadership is a motivator of beneficial behavior, such as innovation. Researchers also stated that an altruistic leader creates a compassionate and flexible work environment, which may encourage healthy behaviors among employees. This could also prevent knowledge from hiding among the employees for the betterment of organizations. So, according to supportive literature, it could be claimed that altruistic leadership negatively affects KH. Hence, we proposed this hypothesis given below.

**H_2_:** *Altruistic leadership negatively impacts knowledge hiding*

### Workplace Friendship Has a Positive Association With Positive Emotions

Despite the difficulties, the employees of the organization' can be seen as providing a variety of good emotional benefits such as satisfaction or pride (Sturm et al., 2022). Positive emotions in the workplace have been demonstrated to be critical for a variety of desirable job consequences both at the individual and organizational levels. Van den Broeck et al. (2021) have utilized psychological theory to demonstrate that positive emotions have always had an impact on cognitive functioning styles and work-related behavior (Fredrickson, 2001). Workplace friendship is defined as “a type of non-coercive interpersonal communication built on the voluntary premise.”

Workplace friendship seems to have a self-regulating impact as an emotional attachment variable and has already been widely explored in the disciplines of psychology and organizational behavior. To be more precise, workplace friendship is a friendly connection that develops among employees based on official work interaction and has voluntary, non-exclusive, as well as personal qualities (Butt et al., 2021a). Individuals that have a high level of workplace friendship will be more inclined to share perks and resources (Yu et al., 2021). Workplace friendship not only addresses an individual's emotional requirements at work but also promotes information exchange and mutual assistance among coworkers. As a result, we propose the following hypothesis:

**H_3_:** *Workplace friendship has a positive effect on positive emotions*

### Altruistic Leadership Has a Positive Effect on Positive Emotions

According to the leader-member exchange hypothesis (LMX), leaders treat followers differently depending on the type of transaction. These interactions result in a variety of high-quality connections (Dust et al., 2021). A huge number of studies have been written about the relationship between leadership and outcomes. The current study investigates altruistic leadership, a type of constructive leadership (Butt, 2019). This type of leadership might seek to avoid KH behavior in workers. Altruistic leadership, also known as human-centered leadership, refers to a leader's behavior that exhibits altruistic compassion for his employees by putting their interests ahead of his own (Salas-Vallina and Alegre, 2018).

In contradiction to other human-centered leadership styles (such as inspiring, real, moral, as well as servant leadership), altruistic leadership focuses on workers' needs and compromises self-interest to serve employees (Hedlund-de Witt, 2012). Altruistic leaders aim to encourage their colleagues to build their devotion and passion by tapping on their subordinates' ideas and emotions (Paul and Jena, 2022). Team members will see such action as “foregoing” their interests to enhance productivity for the greater good of the business. Altruistic leadership is essential for instilling beneficial behaviors in employees, such as job satisfaction, conversational humor, as well as innovation (van Dierendonck and Patterson, 2015). Therefore, the authors proposed this hypothesis based on previous studies.

**H_4_:** *Altruistic leadership has a positive effect on positive emotions*

### The Mediating Role of Positive Emotions

Employees who concentrate on workplace friendships to promote the firm's interests are more likely to be honest with one another, which discourages detrimental work attitudes such as information concealing and knowledge retaining (Rhee and Choi, 2017). Similarly, firms may encourage workers to form professional friendships since such bonds generally result in strong dedication, which may serve as a valuable deterrent to KH behavior (Jiang et al., 2019). According to a new study, while KH is a severe problem in many firms, managers may limit the negative effects by cultivating personal interactions (Zhang and Min, 2019). We also believe that people who work together again and know one another personally are more likely to be helpful in tough times.

As a result, people may be unwilling to engage in KH behavior. Unfortunately, all of this is based on assumptions, and no experimental data exists to back up these claims (Boz Semerci, 2019). Businesses and organizations have suffered significant losses as a result of KH's actions (Hedlund-de Witt, 2012). Furthermore, no attempt was made to indicate what firms may do to prevent knowledge concealment (Butt et al., 2021b). To address this gap, our research has concentrated on the roles of altruistic leadership and workplace friendships in preventing or mitigating the detrimental impacts of KH behaviors in businesses (Connelly et al., 2012). By examining the correlations between altruistic leadership, workplace behaviors, good emotions, and KH behaviors, this study attempted to address a few issues concerning preventative strategies for KH behaviors in businesses (Salas-Vallina and Alegre, 2018).

The direct links between altruistic leadership and KH behavior revealed a negative but significant relationship, showing that this type of leadership was beneficial in reducing the consequences of KH behaviors among employees (Arain et al., 2020). The likely reason is that this type of leadership itself is an optimistic leadership under which the leader compromises he wants to pay attention to the needs of his workers, which in turn boosts employee morale and motivates them to work tirelessly for their companies.

Positive emotions were also found to be directly associated with the leadership style (Zhou and Wu, 2018). Although it was clear that altruistic leadership promotes pleasant feelings among employees, which aids in the prevention of KH behaviors among coworkers (Butt and Ahmad, 2019). A previous study also discovered a substantial mediation effect between altruistic leadership and KH behaviors, highlighting the significance of preventing KH behaviors among workers. Therefore, previous studies support these hypotheses.

**H_5_:** *Positive emotions mediate the relationship between workplace friendship and knowledge hiding*

**H_6_:** *Positive emotions mediate the relationship between workplace friendship and knowledge hiding*

**Figure 1:**
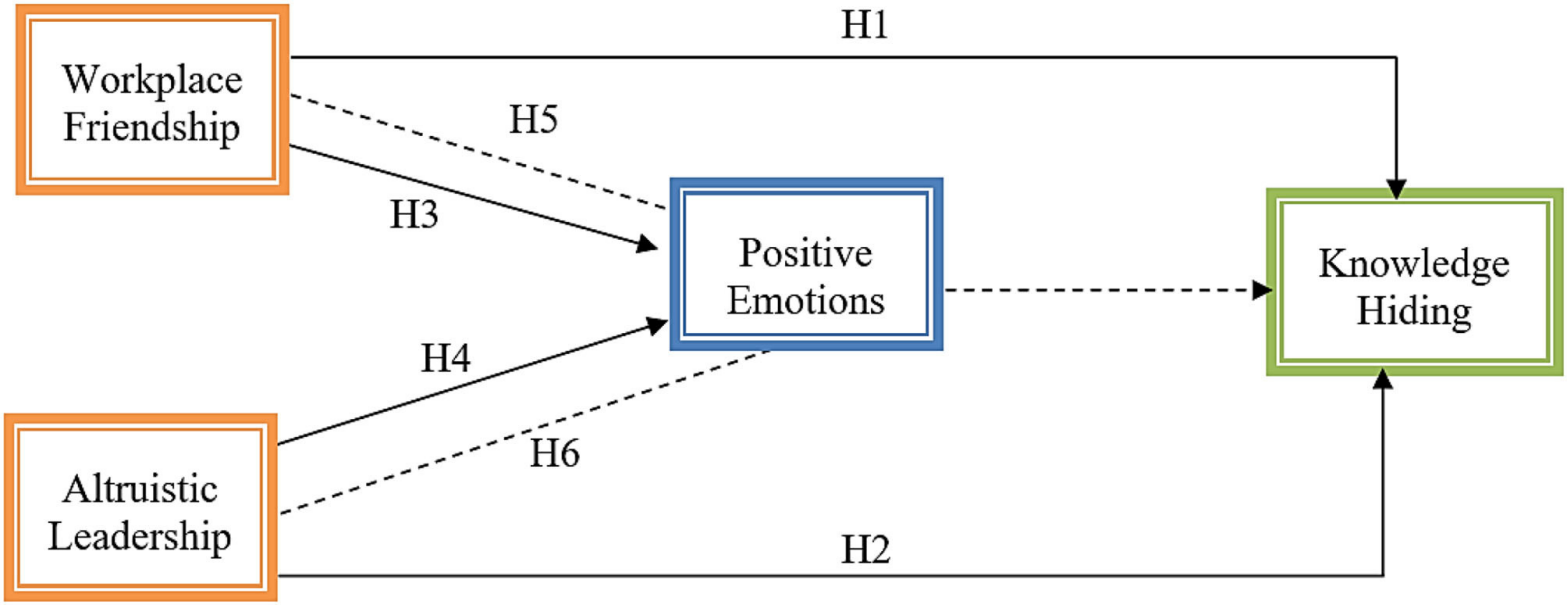
Research Framework.

The following study model (see Figure 1) has been established based on the above literature support and hypothesis development.

## Methodology

A quantitative research design has been chosen for the present study as it is based on the hypothesis testing for primary data, hence following the positivism approach (Nawaz et al., 2022). In this study, the hypotheses have been formulated through the intense review of literature which will be tested statistically for the acceptance or rejection. The study has posed different hypotheses regarding the effect of workplace friendship, altruistic leadership, and positive emotions on the knowledge hiding behavior. The study further checks the mediating role of positive emotions in the relationship of workplace friendship, altruistic leadership, and knowledge hiding behavior. Since the broader concepts have been confined to the theory in the form of hypotheses, hence underpin the deductive way of research (Agarwal et al., 2020).

The target population chosen in this study is the employees of the government sector in China. This particular population has been chosen because they are a more proficient sector in China (Ren et al., 2021). The researcher must select the right population for the study to generate credible, accurate, and bias-free results (Seo et al., 2021). The sampling technique used to select the sample size is convenience sampling. Convenience sampling has been used to select the sample (Etikan et al., 2015), because it is cost-effective, and the respondents are readily available. Another advantage of convenience sampling is that if the respondents are not willing to participate in the study, there are other options as well to include other potential participants.

Literature has proposed different techniques for determining the appropriate sample sizes, however, there is no consensus on the accuracy of the exact sample size (Stratton, 2021). According to (Roscoe et al., 2018) a general rule of thumb is that a sample size between 30 and 500 participants is appropriate and sufficient for conducting a research survey. Therefore, the sample size taken in this study is 496. This is a cross-sectional study as the data was collected in one episode only. The questionnaires had been distributed to the possible respondents and the questionnaire had been explained to them to avoid any misunderstanding regarding the items. Further, it was a self-administered survey for the available respondents, while the rest of the questionnaires had been dropped at the office coordinators. These had been collected a week later, and those left were collected another week later. The total number of questionnaires distributed was 600 while the usable questionnaires received by the researcher were 496. The preliminary screening was done by reading the individual questionnaires. In this process, the questionnaires with spurious responses were deleted.

The technique used for data analysis is partial least square structural equation modeling. This technique gives the researcher liberty of small sample sizes, and simultaneous regression among all variables considering a variable as dependent and independent in another simultaneous relationship (Hubona, 2021). This generates the results based on covariance among the variables and without any compulsion of being normally distributed (Hair et al., 2013). Through the partial least square structural equation modeling, the data is analyzed using the measurement model and the structural model. In the measurement model, the data is screened for the validity and reliability of the scales used for the collection of data. In the structural model, the hypotheses are checked using the *t*-statistics, *p-*values and beta values.

### Instrument Development

The questionnaire used in the study was designed on a 5-point Likert scale (Adam, 2020), which ranged from the responses 1–5 (strongly disagree to strongly agree). The scales for each variable in this study have been adapted from past studies that had an acceptable Cronbach and average variance extracted. The independent variable workplace friendship has been taken from Hsiao-Yen et al. (2009) which consisted of 12 items. The sample items included (1) I have the opportunity to get to know my coworkers, (2) I can collaborate with coworkers to address problems, and (3) In the company, I have the opportunity to privately chat with 260 bureaucracy and workplace friendship with coworkers. The second independent variable of altruistic leadership consists of five items taken from Barbuto and Wheeler (2006). The mediating variable, positive emotions, consisting of four items has been taken from Lundqvist and Kenttä (2010). Similarly, the dependent variable of the present research is knowledge hiding behavior. This variable consisted of three items that were taken from Peng (2013). The Cronbach alpha and convergent validity of the scales are given in **Table 2**. The responses obtained from the data collection have been analyzed using the software Smart-PLS.

### Demographic Analysis

The demographic profile of the respondents is sorted based on frequency and percentage. The age factor has been divided into four categories like age 20–30, 31–40, 41–50, and above 50. The highest number of respondents belonged to the category above 50, then the 20–30 age category, and so on. For the factor of gender, males and females are found somewhat equal in this study. The factor of education was divided into three categories, bachelor's (38.9% of respondents belonged to this category), master's (40.1% of respondents belonged to this category), and PhD and others (20.9% of respondents belonged to this category). Demographic results are shown in Table 1.

**Table 1:** Demographics analysis.

**Demographics**	**Frequency**	**Percentage**
**Age (years)**		
20–30	125	25.2%
31–40	98	19.7%
41–50	84	26.0%
Above 50	189	38.1%
**Gender**		
Male	237	47.7%
Female	259	52.2%
**Education**		
Bachelors	193	38.9%
Masters	199	40.1%
PhD and others	104	20.9%

## Data Analysis and Results

Using SMART-PLS software, the results are generated in two stages. First, the measurement model stage, and second, the structural model stage. With the help of the measurement model, the validity and reliability of the data are checked, while the structural model helps in supporting or not supporting the hypotheses generated. The measurement model checks the reliability and validity of the data based on the preliminary screening tests. These tests include factor loadings, inflation factors, average variance extracted, Heterotrait mono trait ratio, and Fornell and Larcker criteria. The hypotheses, on the other hand, were checked through the second stage of PLS which is structural model measurement. It is checked through *t*-statistics, beta values, and *p*-statistics.

### Measurement Model

**Figure 2:**
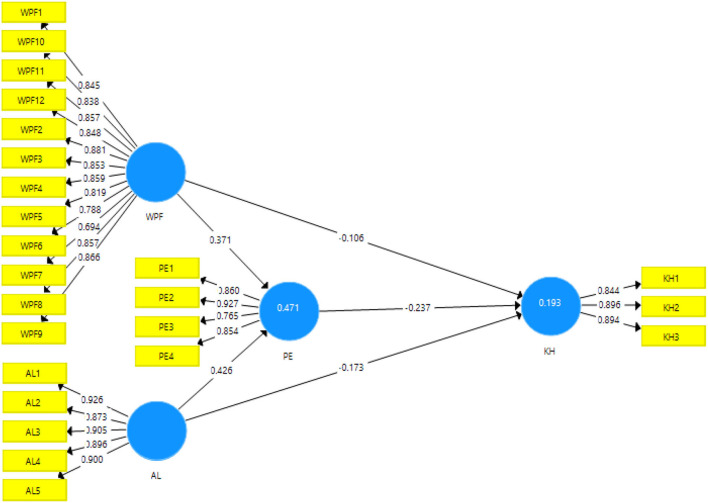
Output of the measurement model. WPF, Workplace friendship; AL, Altruistic leadership; PE, Positive emotions; KH, knowledge hiding.

The measurement model uses convergent validity and reliability to test the acceptability of the data obtained in the survey. The statistics used for confirming the convergent validity are factor loadings, average variance extracted, Cronbach, and composite reliability (Troiville et al., 2019). The output for measurement is given in Figure 2.

The factor loadings of the items used in the study should be above 0.7 (Lambert et al., 1991). The present study meets the criteria by showing values above the threshold. The variable of altruistic leadership showed the factor loadings for all its six items to be above 0.8 which confirms the convergent validity of this variable. Similarly, the knowledge hiding variable shows the minimum factor loading of 0.8 which is also above the threshold mentioned in the literature. Variable Positive emotions also met the criteria of significant factor loadings by showing the minimum loading of item PE3 which is 0.765.

The variable of workplace friendship showed the factor loadings above 0.7 except for the item WPF9 which is 0.788 and also supposedly meets the criteria. Cronbach alpha and composite reliability have been considered significant and acceptable if their values are above 0.7 (Perry et al., 2005). In this study, the reliability is confirmed since the lowest Cronbach alpha is 0.855 for variable knowledge hiding. As long as the average variance extracted is concerned, the minimum acceptance value is 0.5 indicating that the variance extracted for this particular variable is more than the error (Dash and Paul, 2021). The minimum AVE in the study is for variable workplace friendship which is 0.697 yet meets the acceptance criteria. The results for the convergent validity and reliability can be seen in Table 2.

**Table 2:** Factor loadings cronbach alpha, composite reliability, and AVE.

**Variables**	**Factor loadings**			**Cronbach**	**Composite reliability**	**AVE**
Altruistic leadership	AL1		0.926			
	AL2		0.873	0.941	0.955	0.810
	AL3		0.905			
	AL4		0.896			
	AL5		0.900			
Knowledge hiding	KH1		0.844			
	KH2		0.896	0.855	0.910	0.771
	KH3		0.894			
Positive emotions	PE1		0.860			
	PE2		0.927	0.876	0.914	0.728
	PE3		0.765			
	PE4		0.854			
Workplace friendship	WPF1		0.845			
	WPF2		0.838	0.960	0.965	0.697
	WPF3		0.857			
	WPF4		0.848			
	WPF5		0.881			
	WPF6		0.853			
	WPF7		0.859			
	WPF8		0.819			
	WPF9		0.788			
	WPF10		0.694			
	WPF11		0.857			
	WPF12		0.866			

Discriminant validity helps the researcher to understand if the variable measures the facet that is supposed to be measured (Prochazka and Schweiger, 2018). In this study, two statistical tests have been used which are widely used in research. First of all, the Fornell and Larcker criteria and the HTMT ratio stand for hetero trait mono trait ratio (Franke and Sarstedt, 2019). The Fornell and Larcker criteria table confirms the discriminant validity when each column shows its highest number at the top (Fornell and Larcker, 1981). In this study, columns of all variables show the highest number of their column at the top (see Table 3). Similarly, for the HTMT ratio to confirm the discriminant validity all ratios in the table must be well below 0.85 (Franke and Sarstedt, 2019). In this study, the highest value in the table is 0.674 which means the data confirms discriminant validity (see Table 3).

**Table 3:** Discriminant validity (HTMT Ratio and Fornell and Larcker Criteria).

	**Heterotrait-monotrait ratio (HTMT)**					**Fornell-larcker criterion**			
	**AL**	**KH**	**PE**	**WPF**		**AL**	**KH**	**PE**	**WPF**
**AL**					**AL**	0.900			
**KH**	0.396				**KH**	−0.367	0.878		
**PE**	0.647	0.440			**PE**	0.604	−0.402	0.854	
**WPF**	0.504	0.341	0.612		**WPF**	0.481	−0.325	0.576	0.835

*WPF, Workplace friendship; AL, Altruistic leadership; PE, Positive emotions; KH, knowledge hiding*.

### Structural Model

**Figure 3:**
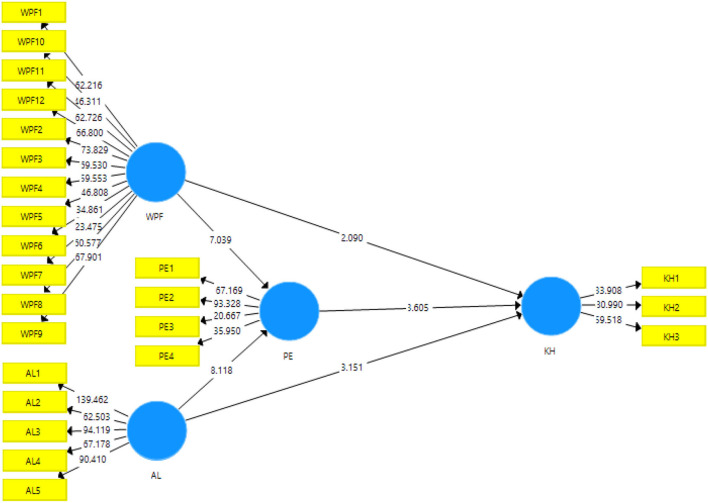
The output of structural model Bootstrapping. WPF, Workplace friendship; AL, Altruistic leadership; PE, Positive emotions; KH, knowledge hiding.

The structural model helps the researcher in understanding the criteria and knows if the hypotheses formulated in the literature are supported by the data or not (Hair et al., 2014; Hubona, 2021). In this study, the bootstrapping command is run with a 95% confidence interval and a sub-sampling of 500 (Yao et al., 2020). The statistics used to confirm the hypothesis are *t*-value, *p*-value, and beta coefficient. Results of the bootstrapping are shown in Figure 3.

In this study, the direct effects among different variables have been checked (see Table 4). The first direct effect of the study explains the first hypothesis i.e., workplace friendship has a negative impact on knowledge hiding behavior. The hypothesis is supported by the data showing beta = −0.108, *t* = 2.0, *p* = 0.037. The second hypothesis is about altruistic leadership having a negative impact on knowledge hiding behavior. This hypothesis is supported by data showing beta = −0.173, *t* = 3.151, *p* = 0.002. The third hypothesis about workplace friendship having an effect on positive emotions is also supported showing beta = 0.372, *t* = 7.03, *p* = 0. Fourth hypothesis about altruistic leadership having an impact on positive emotions beta = 0.427, *t* = 8.11 *p* = 0.

**Table 4:** Direct effects.

**Paths**	***H***	**β**	**SD**	***T*** **-statistics**	***P*** **-value**
**WPF -> KH**	H_1_	−0.108	0.050	2.090	**0.037**
**AL -> KH**	H_2_	−0.173	0.055	3.151	**0.002**
**WPF -> PE**	H_3_	0.372	0.053	7.039	**0.000**
**AL -> PE**	H_4_	0.427	0.053	8.118	**0.000**

*WPF, Workplace friendship; AL, Altruistic leadership; PE, Positive emotions; KH, knowledge hiding. Bold values shows the relationship between the variables*.

In this study, there are two indirect effects which are given in Table 5. The first indirect effect is about the mediating role of positive emotions between the relationship of workplace friendship and knowledge hiding. This hypothesis is supported showing *t* = 3.108, *p* = 0.002. The second indirect effect is about the mediating role of positive emotions between the relationship of altruistic leadership and KH. This hypothesis is supported by showing *t* = 3.462 and *p* = 0.001.

**Table 5:** Indirect effects.

**Paths**	***H***	**β**	**SD**	***T*** **-statistics**	***P*** **-value**
**AL -> PE -> KH**	H_5_	−0.101	0.029	3.462	**0.001**
**WPF -> PE -> KH**	H_6_	−0.088	0.028	3.108	**0.002**

*WPF, Workplace friendship; AL, Altruistic leadership; PE, Positive emotions; KH, knowledge hiding. Bold values shows the relationship between the variables*.

## Discussion

This research focused on identifying the role of some preventive measures to limit or eliminate the knowledge hiding behaviors from the employees. Previously, Afshan et al. (2022) found that followers have an impact on supervisors by influencing their behaviors for knowledge management at an organizational level. It is also proven in some of the previous studies that knowledge hiding is directly related to lessened or disturbing performances of the enterprises. These investigations also noted that KH behaviors at times reduce the innovativeness of employees which prevents them from progressing positively. This also leads to self-deviance from the targets of the organizations they are working for (Ren et al., 2021). There was a strong need to identify and evaluate the factors which could lead to preventing such KH aptitudes from hindering the progress of organizations (Hubona, 2021). The current research looked into the factors which could help in preventing the KH behaviors of the organizations such as workplace friendships and altruistic leadership.

This study also tried to find out the impact of workplace friendships and altruistic leadership on positive emotions. The supportive and mediating role of positive emotions were also tested in this research. The direct effects of workplace friendships on KH behavior showed that there was a negative relationship between both. It indicated that such friendships at the workplace could be a helpful tool in preventing the negative KH behaviors in the organizations which lead to reduced efficiency of the organizations. The possible reasoning for this result lies in the fact that if workers at the workplace are not socially in contact with each other or have common interests then they could not do well. This thing will stop them from sharing knowledge with colleagues resulting in no cooperation and motivation for each other. Previously, Hubona (2021) reported that if colleagues were not connected then it promoted the KH behavior among themselves and reduced their engagement to work performance.

Similar kinds of results were also obtained by some other researchers which indicated that workplace friendships were necessary to prevent such KH behaviors which cause poor performance at a large level (Dust et al., 2021). It was also observed previously that if colleagues cooperate then it could help in developing a strong association between them and also help in preventing KH behaviors (Arain et al., 2022). The results of the relationship between workplace friendships and positive emotions indicated that such friendships also lead to the development of positive emotions among colleagues. Such emotions are the direct output of friendships among colleagues. Previously, no scholar has investigated this kind of association between workplace friendship and the positive emotions of the employees.

The reason for these kinds of results is probably due to the connectedness of the employees with each other. The direct relationship of altruistic leadership with KH behavior indicated that there was a negative but significant association between both indicating that this kind of leadership helped mitigate the effects of KH behaviors among the employees. The possible reason lies in the fact that this kind of leadership is itself an optimistic leadership in which the leader sacrifices his own needs and pays heed to the employees' needs which in turn boosts the morale of the employees to work devotedly for their organizations (Fehlings, 2020). It is also evident that such kind of leadership helps in shaping the behaviors of employees which is a psychological attribute associated with employees.

The literature also suggested that leadership plays an important role in the behavior molding of employees at an organizational level. Previously, most of the research only focused on defining leadership and its styles, which undoubtedly helped in understanding the significance of leadership. A few investigations expressed that certain leadership styles were directly related to the components of behavioral changes among the subordinates which is the psychological approach to enhance the efficiency of workers and obtain good performance at the organizational level (Yukl et al., 2019). The relationship between altruistic leadership and positive emotions also proved to be positively significant. Some of the past researchers indicated that this kind of leadership is capable of developing positive emotions among the employees (Belwalkar et al., 2018).

It was also observed in research those positive emotions were directly related to the style of leadership (Dasborough, 2006). Positive emotions are also the psychological aspect of employees' performance at large. Once, the leadership is working in the right direction, it helps in directing the emotions of employees positively for the betterment of the organization. It was also evident that altruistic leadership develops positive emotions in the workers which in turn helps in preventing KH behaviors among the colleagues. Leadership is not only limited to work-related attributes, but it also helps in improving the psychological aspects of the employees. Such motivated employees influenced by positive leaders would do their work honestly when provided with psychological comfort and it boosts their efficiency (Yukl et al., 2019).

Current research also found that mediating impact between altruistic leadership and KH behaviors was significant indicating the aiding role of positive emotions in preventing KH behaviors among the workers. As discussed earlier, positive emotions of employees are more of a psychological approach meaning that they are more to do with the psychology of employees which in turn shapes the productive behaviors. The current results are also supported by the research of Liden et al. (2013). The mediating effect of positive emotions was also tested between workplace friendships and KH behaviors. Results indicated that positive emotions also helped in preventing the KH behaviors in direct relation to workplace friendships. A similar kind of mediating role of positive emotions was also presented Belwalkar et al. (2018). The reason for such results could be the connectedness of the workers at the workplace who support each other in achieving the shared goal.

### Practical Implications and Theoretical Contribution

There are a few implications of the study that can be availed by the organizations to improve their organizational environment. Firstly, organizations must encourage leadership that is empathetic toward their employees and provide a conducive environment that gives positive vibes to each other. Secondly, workplace friendships should be promoted to generate positive emotions among the employees which results in a decrease in the knowledge hiding behaviors in the organizations. Thirdly, when the organizations ensure workplace friendships and empathy in the leadership, it helps in preventing the knowledge hiding behaviors in the organization at any level.

The theoretical consideration of the study offers an extension of literature in the field of organizational behavior and psychology. First, the present study has tested that workplace friendship harms knowledge hiding behavior. Second, the presence of altruistic leadership has also been found to decrease knowledge hiding behavior. Thirdly, the presence of positive emotions among employees helps reduce the knowledge hiding behavior.

### Limitations and Directions for Future Studies

There are some limitations to this study. First of all, the study has used a sample of employees of the government sector of China which gives limited exposure to the data collection. Hence, it is important to use probability sampling to get more generalized results. Moreover, the present theoretical framework included only workplace friendship and altruistic leadership. Another reason for incorporating non-probability sampling is the availability of limited time. This is because of the current pandemic people are hardly available, and those available tend to avoid being part of research because a lot of research has been conducted recently on the pandemic (Chesbrough, 2020; Commodari and La Rosa, 2020; Farooq et al., 2020; Ratten, 2020; Yousaf et al., 2020).

Future studies are recommended to conduct time series analysis by conducting the data at different points of time and checking if there is any deviation in the results. Furthermore, it is also recommended that studies are conducted in different sectors of China to see if the knowledge hiding preventive behaviors is observed based on altruistic leadership and promoting workplace friendships. The sample size had been sufficient (*N* = 496) for structural equation modeling to be applied using other variance-based approaches. They are claimed to generate more accurate and less biased results every time they are employed. Therefore, it is suggested that future studies use variance-based software to see if it shows any deviation in the results. It is suggested that this study uses other leadership styles as well to understand if it helps in preventing knowledge hiding behaviors. Further, it will also be interesting to include the moderation effect to understand if it disturbs these relationships. The potential moderating variables can be task performance, workplace deviance, leader-member exchange, etc.

## Conclusion

Knowledge sharing behavior has been the center of attention for more than a decade; however, knowledge hiding and its preventive measures have a gap in the literature. This study has investigated the impact of workplace friendship and altruistic leadership in preventing knowledge hiding behavior in organizations. The results of the study have indicated that workplace friendship and altruistic leadership have been found to play a significant role in preventing knowledge hiding behavior. Further, positive emotions have also been found to help the relationship between workplace friendship and knowledge hiding prevention. Similarly, it also augments the relationship between altruistic leadership and knowledge hiding prevention. The study offers certain implications for the organization by promoting workplace friendships to generate positive emotions among the employees which results in a decrease in the knowledge hiding behaviors in the organizations.

## Data Availability Statement

The original contributions presented in the study are included in the article/supplementary material, further inquiries can be directed to the corresponding author/s.

## Ethics Statement

The studies involving human participants were reviewed and approved by Nanjing University, China. The patients/participants provided their written informed consent to participate in this study. The study was conducted in accordance with the Declaration of Helsinki.

## Author Contributions

YH: supervision, conceived, designed, and wrote the article. XW: data collection. All authors read and agreed to the published version of the manuscript.

## Conflict of Interest

The authors declare that the research was conducted in the absence of any commercial or financial relationships that could be construed as a potential conflict of interest.

## Publisher's Note

All claims expressed in this article are solely those of the authors and do not necessarily represent those of their affiliated organizations, or those of the publisher, the editors and the reviewers. Any product that may be evaluated in this article, or claim that may be made by its manufacturer, is not guaranteed or endorsed by the publisher.
